# First-Generation Antipsychotic Haloperidol Alters the Functionality of the Late Endosomal/Lysosomal Compartment *in Vitro*

**DOI:** 10.3390/ijms17030404

**Published:** 2016-03-18

**Authors:** Alberto Canfrán-Duque, Luis C. Barrio, Milagros Lerma, Gema de la Peña, Jorge Serna, Oscar Pastor, Miguel A. Lasunción, Rebeca Busto

**Affiliations:** 1 Servicio de Bioquímica-Investigación, Hospital Universitario Ramón y Cajal, IRyCIS, 28034 Madrid, Spain; alberto_canfran@hotmail.com (A.C.-D.); milalermafernandez@yahoo.es (M.L.); gema.delapena@hrc.es (G.d.l.P.); 2 Unidad de Neurología Experimental, Hospital Universitario Ramón y Cajal, IRyCIS, 28034 Madrid, Spain; luis.c.barrio@hrc.es; 3 Servicio de Bioquímica-Clínica, Hospital Universitario Ramón y Cajal, IRyCIS, 28034 Madrid, Spain; jserna_87@hotmail.com (J.S.) oscar.pastor@salud.madrid.org (O.P.); 4 CIBER Fisiopatología de la Obesidad y Nutrición (CIBEROBN), Instituto de Salud Carlos III (ISCIII), 28029 Madrid, Spain

**Keywords:** antipsychotic, haloperidol, cholesterol, intracellular lipid traffic, late endosome/lysosome, pH

## Abstract

First- and second-generation antipsychotics (FGAs and SGAs, respectively), have the ability to inhibit cholesterol biosynthesis and also to interrupt the intracellular cholesterol trafficking, interfering with low-density lipoprotein (LDL)-derived cholesterol egress from late endosomes/lysosomes. In the present work, we examined the effects of FGA haloperidol on the functionality of late endosomes/lysosomes *in vitro*. In HepG2 hepatocarcinoma cells incubated in the presence of 1,1′-dioctadecyl-3,3,3,3′-tetramethylindocarbocyanineperchlorate (DiI)-LDL, treatment with haloperidol caused the enlargement of organelles positive for late endosome markers lysosome-associated membrane protein 2 (LAMP-2) and LBPA (lysobisphosphatidic acid), which also showed increased content of both free-cholesterol and DiI derived from LDL. This indicates the accumulation of LDL-lipids in the late endosomal/lysosomal compartment caused by haloperidol. In contrast, LDL traffic through early endosomes and the Golgi apparatus appeared to be unaffected by the antipsychotic as the distribution of both early endosome antigen 1 (EEA1) and coatomer subunit β (β-COP) were not perturbed. Notably, treatment with haloperidol significantly increased the lysosomal pH and decreased the activities of lysosomal protease and β-d-galactosidase in a dose-dependent manner. We conclude that the alkalinization of the lysosomes’ internal milieu induced by haloperidol affects lysosomal functionality.

## 1. Introduction

People with severe mental illness, including schizophrenia and related psychotic disorders, experience a two to three times higher mortality rate than the general population [1]. Their treatment with antipsychotic medication is a contributory factor, not least because of the metabolic side effects [2,3]. Two classes of antipsychotic drugs, typical or first-generation antipsychotics (FGAs) and atypical or second generation antipsychotics (SGAs) are recognized, which therapeutic efficacy depends on the antagonism of D_2_ dopamine receptors [4]. FGAs exhibit greater affinity for D_2_ receptors than SGAs and lower affinity to serotonergic receptors, among other neurotransmitter receptors [4], exert deleterious effects when administered for long periods of time. The incidence of neurological side effects (extrapyramidal symptoms or movement disorders) is usually higher with FGAs, but both types of drugs may produce metabolic adverse effects [5,6]. Metabolic syndrome is relatively common in people on long-term antipsychotic treatment [7,8,9].

Plasma low-density lipoprotein (LDL) is the main source of cholesterol for cells in humans. LDL is taken up by cells via the LDL receptor present in the plasma membrane [10]. After endocytosis, the LDL lipids are hydrolyzed in lysosomes and free (unesterified) cholesterol is then transported to endoplasmic reticulum (ER) and other intracellular organelles. In the ER, cholesterol is sensed by the sterol regulatory element-binding protein (SREBP)-Scap-Insig system, which triggers a homeostatic response covering the whole lipid metabolism [10]. The excess of free cholesterol is esterified by lecitin colesterol acil transferase (LCAT) and stored in the cytoplasm as lipid droplets [10].

Antipsychotics have been shown to affect lipid homeostasis [11,12,13,14,15]. FGAs and SGAs reduce cholesterol biosynthesis by inhibiting the activities of several enzymes in the final steps of the pathway, which leads to the accumulation of sterol intermediates. The actual enzyme inhibited depends on the specific drug and the dose used [11,12,14,16]. As a result, the sterol composition of membrane microdomains is changed and the signaling of receptors present in those domains is altered, as shown for insulin and somatostatin receptors [11,16].

Antipsychotics also affect the intracellular cholesterol trafficking by interfering with the cholesterol egress from the endosomal/lysosomal compartment to the ER [11,12,14]. This effect is in line with the upregulation of SREBP observed in cells treated with antipsychotics [14,17]. In this way, exposure to antipsychotics secondarily leads to the stimulation of LDL endocytosis, thus aggravating the intracellular accumulation of LDL-derived lipids, and also stimulates fatty acid and triacylglycerol biosynthesis [14].

The ionic composition of different intracellular compartments differs from the cytoplasm, and is unique and characteristic for each of them. There is a pH gradient which falls from the ER (pH ~7.1), to the Golgi (pH ~6.2–7.0) and them to the secretory organelles (pH ~5.0); and from the early endosome or late endosome (pH ~6.5) to lysosomes (pH ~4.5) [18,19]. This ionic composition is important for the function of the organelles. Thus, the acidic pH of the lysosomes is necessary for enzymatic activity, receptor recycling to the plasma membrane, and fusion of lysosomes with other organelles and the cell membrane itself [20,21].

In this study, we aimed to analyze the effects of FGA haloperidol on the functionality of late endosomes/lysosomes *in vitro*. We show that haloperidol increases the alkalinity of lysosomes and affects the activity of enzymes located in this cellular compartment.

## 2. Results

### 2.1. Effects of Haloperidol on the Endo-Lysosomal Compartment

In this study, we chose the HepG2 cells for their active lipid metabolism and because of the involvement of liver in the metabolism of antipsychotics. We previously reported that both FGAs and SGAs interfere with LDL-derived cholesterol egress from the late endosomes/lysosomes and thereby induced lipid accumulation in the endosomal/lysosomal compartment [11,14,22].

**Figure 1:**
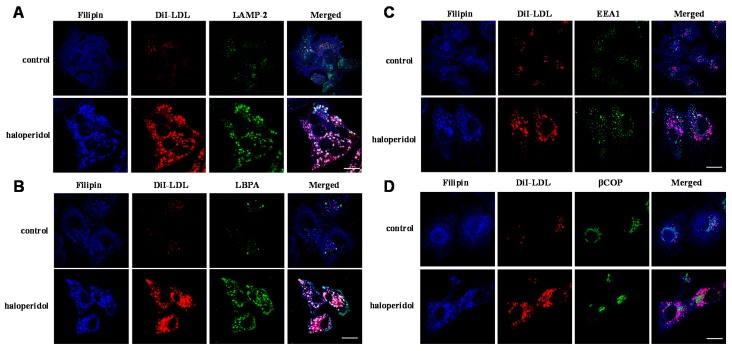
Effects of first-generation antipsychotic (FGA) haloperidol on the accumulation of low-density lipoprotein (LDL)-derived lipids in the late endosomal/lysosomal compartment. Untreated HepG2 cells (control) and cells treated for 16 h with 10 µM haloperidol were incubated with DiI-LDL (30 µg/mL of cholesterol), then were fixed and stained with filipin (free cholesterol): (**A**) cells stained for lysosome-associated membrane protein 2 (LAMP-2) (endo-lysosomes); (**B**) cells stained for LBPA (endo-lysosomes); (**C**) cells stained for early endosome antigen 1 (EEA1) (early endosomes); and (**D**) cells stained for coatomer subunit β (β-COP) (Golgi). (**A**–**D**) Cells were photographed using a confocal microscope. Scale bar, 10 µm. Micrographs from a single representative experiment, of the three independent experiments performed, are shown.

In this study, we sought to characterize the functionality of the late endosome/lysosome compartment in HepG2 cells treated with the FGA haloperidol. HepG2 cells were incubated with 1,1′-dioctadecyl-3,3,3,3′-tetramethylindocarbocyanineperchlorate (DiI)-labeled LDL (30 µg/mL of cholesterol) for 16 h in the absence(control) or the presence of haloperidol (10 µM) and then stained with filipin (for detection of free cholesterol), and anti- lysosome-associated membrane protein 2 (LAMP-2) (a marker of late endosome) or anti-LBPA (lysobisphosphatidic acid or bis(monoacylglyceryl)phosphate (BMP)), a phospholipid present in the internal membranes of late endosome [23] and examined by confocal microscopy. As shown in Figure 1A, treatment with haloperidol caused an increase in bright perinuclear granules positive for LAMP-2, with increased content of both free cholesterol (filipin) and DiI, all indicating their association with late endosome/lysosomes. Similarly, cells treated with haloperidol exhibited intense labeling for LBPA in vesicles positive for filipin and containing DiI (Figure 1B). These results show that the FGA haloperidol provokes the enlargement of the late endosomal/lysosomal compartment.

Furthermore, treatment with haloperidol did not alter the distribution of EEA1, a marker of early endosomes (Figure 1C). Indeed, in haloperidol-treated treated cells, organelles heavily loaded with DiI were not stained for EEA1. This indicates that LDL-derived lipids accumulated in late endosomes/lysosomes and that traffic through the early endosomal compartment was not affected by the antipsychotic. Finally, haloperidol did not perturb the Golgi apparatus (Figure 1D), since it did not change the distribution of the specific marker β-COP.

### 2.2. Effects of Haloperidol on pH Lysosomal

**Figure 2:**
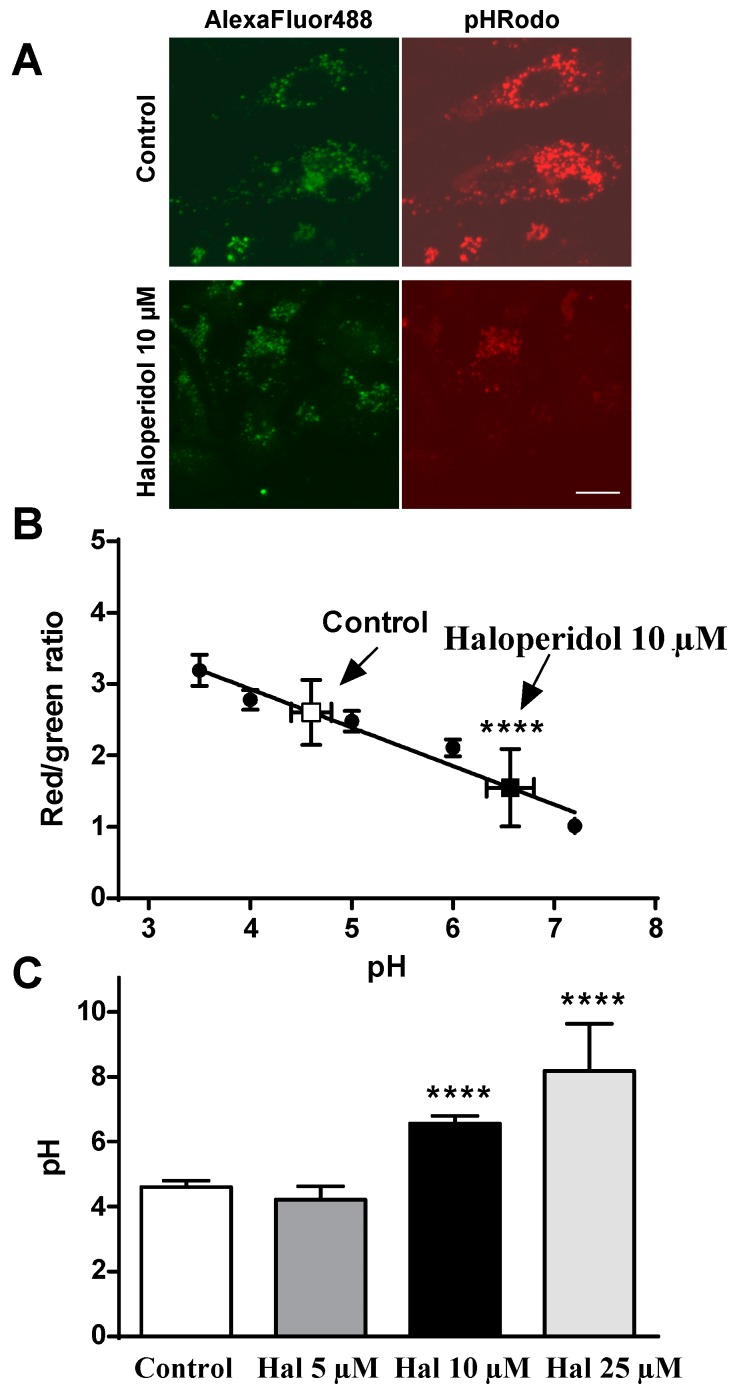
FGA haloperidol increases the lysosomal pH. HepG2 cells were incubated with LDL. (30 µg/mL of cholesterol) for 12 h in the absence (control) or the presence of haloperidol (Hal) (5, 10 or 25 µM) and pH-sensing fluorochromes Alexa Fluor 488-dextran and pHrodo-dextran. (**A**) Sample confocal images of control and 10 µM haloperidol treated culture dishes showing emission reduction of pH sensitive pHrodo (in red) relative to pH insensitive Alexa Fluor probe (in green). Scale bar, 5 µm. (**B**) Standard curve to calculate endogenous lysosomal pH where values of the red:green fluorescence ratio corresponding to control and haloperidol at 10 µM are indicated. (**C**) Effect of increasing concentrations of haloperidol on lysosomal pH. Results are mean ± SEM (*n* = 3–15). Statistical comparisons shown are haloperidol versus control (**** *p* <0.0001).

Since haloperidol affected the intracellular lipid trafficking especially at the level of the late endosomal/lysosomal compartment, we measured the effect of this drug on the pH of late endosomes/lysosomes. For this, HepG2 cells were treated with increasing concentrations of haloperidol (0, 5, 10 and 25 µM) for 12 h and the pH was measured using a method based on confocal microscopy, by staining the lysosomes with Alexa Fluor 488-dextran and pHrodo-dextran as a pH sensor. We observed that the lysosomal pH of control cells was about 4 (Figure 2), which is in agreement with the pH reported by others for this compartment [18,19,20]. Treatment with haloperidol increased the lysosomal pH in a dose-dependent manner, an effect that was highly significant (*p <* 0.0001) with doses of 10 (6.56 ± 0.24, mean ± SEM, *n* = 15) and 25 µM (8.18 ± 1.46), but not with 5 µM (4.21 ± 0.41) (Figure 2). The alkalinization of lysosomes induced by haloperidol may affect the functionally of these organelles.

### 2.3. Effects of Antipsychotic on Lysosome Enzyme Activities

**Figure 3:**
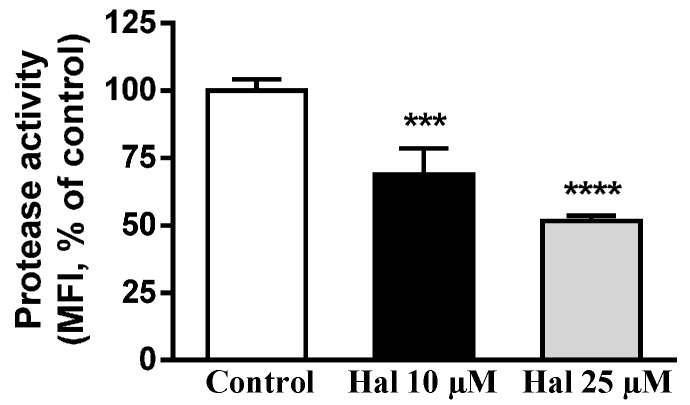
Haloperidol decreases the activity of lysosomal proteases. THP-1 macrophages cells incubated in lipoprotein-deficient serum (LPDS) medium with LDL were kept untreated (control) or treated with haloperidol (10 and 25 µM) for 30 min, then DQ-BSA was added for 30 min and analyzed by flow cytometry. Results are mean ± SEM of three independent experiments. MFI, median fluorescence intensity. Statistical comparisons shown are haloperidol *versus* control (*** *p* < 0.001 and **** *p* < 0.0001).

The above-mentioned mentioned results encouraged us to study whether treatment with haloperidol affected the lysosomal protease activities. To perform this study we used DQ™ BSA (DQ-BSA), a BODIPY^®^ FL-labeled albumin derivative that initially does not fluoresce. After binding to the Fc receptor, the complex is internalized and after hydrolysis of the protein emits fluorescence [24]. For this study we used THP-1 cells differentiated to macrophages, because HepG2 cells do not express Fc receptors. THP-1 macrophages maintained in lipoprotein-deficient serum (LPDS) medium with LDL were kept untreated (control) or treated with haloperidol (10 and 25 µM), then the cells were exposed to DQ-BSA and finally cells were analyzed by a FACScan-Flow cytometry system. As shown in Figure 3, treatment with haloperidol dose-dependently decreased the appearance of DQ-BSA-fluorescence, indicating that lysosomal protease activity was reduced.

**Figure 4:**
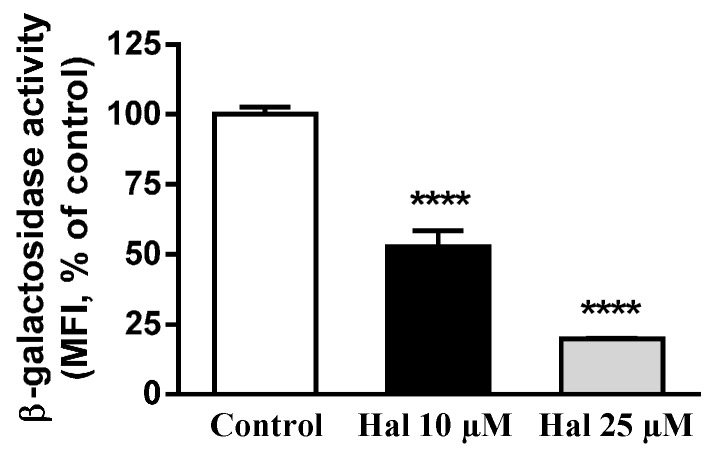
Haloperidol decreases lysosomal β-galactosidase enzyme activity. HepG2 cells incubated in LPDS medium with LDL were kept untreated (control) or treated with haloperidol (10 and 25 µM) for 2 h, then incubated with C_12_FDG for 4 h and analyzed by flow cytometry. Results are mean ± SEM of three independent experiments. MFI, median fluorescence intensity. Statistical comparisons shown are haloperidol *versus* control (**** *p* < 0.0001).

Lysosomal β-d-galactosidase is a hydrolase that cleaves the terminal β-galactose from ganglioside substrates and other glycoconjugates. To assay this activity we used 5-dodecanoylaminofluorescein di-β-d-galactopyranoside (C_12_FDG), a fluorogenic substrate for β-d-galactosidase, which permeates membrane and is non-fluorescent before hydrolysis; after hydrolysis of the galactosyl residues, the compound becomes fluorescent and remains confined within the cell. HepG2 cells maintained in LPDS medium with LDL were kept untreated (control) or treated with haloperidol (10 and 25 µM) and exposed to C_12_FDG ultimately being analyzed by flow cytometry. As shown in Figure 4, treatment with 10 and 25 µM haloperidol strongly decreased cell fluorescence, indicating that haloperidol inhibits lysosomal β-d-galactosidase activity in a dose-dependent manner.

Taken together, the results show that FGA haloperidol induces abnormal structures that exhibit late endosomal markers like LAMP-2 and LBPA, without perturbing the early endosomal or the Golgi compartments. These structures accumulate LDL-derived lipids under the influence of haloperidol. Treatment with the antipsychotic alkalinizes the intralysosomal milieu and decreases the activity of lysosomal proteases and β-d-galactosidase. Therefore, treatment with haloperidol affects lysosomal functionality in cultured cells.

## 3. Discussion

Treatment with antipsychotics is often accompanied by metabolic side effects [5]. Both classes of antipsychotics have been shown to inhibit cholesterol biosynthesis and to cause the accumulation of lipids in late endosomes/lysosomes in different cell lines [11,12,14]. These effects could underlie some of the adverse metabolic effects induced by therapeutic use of these drugs [14]. In this study, we further characterized the effects of FGA haloperidol on the functionality of the late endosomal/lysosomal compartment in human cell lines.

We have shown herein that haloperidol induced the accumulation of LDL-derived lipids in vesicles expressing LAMP-2 and containing LBPA, whilst the early endosomal and the Golgi compartments remained unperturbed. This confirmed and extended previous results showing that treatment with antipsychotics inhibits the egress of LDL-lipids from lysosomes [11,14,25]. Furthermore, we report for the first time that haloperidol can provoke lysosomal alkalinization and decrease the activity of lysosomal enzymes, such as protease and β-d-galactosidase. These results demonstrate that haloperidol treatment impaired lysosomal functionality.

We first studied the changes in the late endosomal/lysosomal compartment in HepG2 cells by immunocytochemistry. We observed that haloperidol promoted the accumulation of LDL-derived lipids in perinuclear vesicles that were positive for LAMP-2 and LBPA. This confirmed and extended previous results showing that treatment with antipsychotics enlarges the late endosomal/lysosomal compartment and inhibits the egress of LDL-lipids from lysosomes [11,12,14,25]. The diminished availability of cholesterol in the ER explains the upregulation of SREBP and their target genes as shown in previous studies [14,17]. Particularly interesting is the upregulation of LDL receptor, which triggers the increase of LDL endocytosis, thus aggravating the lipidosis induced by antipsychotics [14]. These effects were confined to the endolysosomal compartment; the traffic of LDL through the early endosomal compartment and the Golgi apparatus appeared to be unaffected.

LBPA (or BMP) is a negatively charged glycerophospholipid that is mainly localized in late endosomes/lysosomes [26]. Its unusual structural configuration *sn*-1;*sn*-1’ is thought to play a role in the resistance of LBPA to many phospholipases, and its stability in late endosomes/lysosomes [26]. LBPA is intimately connected with unesterified cholesterol [23,27]. The application of anti-LBPA antibodies has been shown to induce the accumulation of cholesterol in lysosomes [27]. Moreover, in fibroblasts from patients with Niemann-Pick type C, exogenous feeding of LBPA decreases excessive cholesterol storage [23]. The increase of LBPA, as effected by haloperidol treatment, could be thus considered as a compensatory mechanism to accommodate the cholesterol excess, as suggested for other pathological conditions [28].

Lysosomes are responsible for degrading much of the waste material within a cell, and the acidic pH of the lysosomal internal milieu is critical for the activity of numerous lysosomal enzymes. We measured lysosomal pH by a technique based on confocal microscopy, using pHrodo-dextran and Alexa Fluor 488-dextran as probes [29,30]. Treatment of cells with haloperidol noticeably increased lysosomal pH in a dose-dependent way. This is the first time the alkalinization of lysosomes under the influence of FGA haloperidol has been reported. Antipsychotics are cationic amphiphile/amphipath drugs (CAD) positively charged by virtue of an amine group that can be protonated, and display both hydrophilic and hydrophobic properties. It is well-recognized that many amines, including antipsychotics [31,32], have a strong propensity to specifically and substantially accumulate in acidic intracellular compartments, such as lysosomes, through a mechanism referred to as ion trapping [33]. In their unionized form, weak basic amines permeable membrane relatively easily, but membranes are impermeable to their ionized conjugate acidic form [33]. The presence of CADs in the organelles may lead to a neutralization of the lysosomes [34] because of the consumption of free protons in the lumen. Other CADs such as chloroquine (an antimalarial drug) [35], imipramine (antidepressant) [36] or tamoxifen (selective estrogen receptor modulator, SERM) [37], have been shown to raise lysosomal pH. Interestingly, several SERMs have been shown to inhibit several enzymes involved in cholesterol biosynthesis, and to affect the LDL endocytotic trafficking and increase the lysosomal pH [37,38,39,40] in a manner similar to the antipsychotics.

An imbalance of lysosomal lipids has been related to lysosomal pH. In Gaucher’s disease, glucosylceramide appears to modulate endolysosomal pH via cholesterol accumulation [41]. Abnormal lysosomal pH has also been reported in other lysosomal storage disorders and cholesterol loading effectively increased lysosomal pH in normal cells [42]. Finally, sphingomyelin has been shown to inhibit lysosomal Ca^2+^ channels [43]. Therefore the suggestion that lipid accumulation also contributes to the increase in lysosomal pH in cells treated with haloperidol, cannot be ruled out.

Alkalinization of the lysosomal lumen has been shown to decrease the activity of various lysosomal enzymes [44,45,46,47]. We measured the activity of two acidic hydrolases *in situ* by flow cytometry-based methods using specific fluorogenic substrates. The results showed that treatment with haloperidol reduced the activity of both lysosomal protease and β-d-galactosidase in a dose-dependent way. This is an expression of the impairment of lysosomal function brought about by haloperidol.

In HepG2 cells, treatment with haloperidol caused the accumulation of LDL-derived lipids in late endosomes/lysosomes, which was accompanied by increased expression of LAMP-2 and LBPA, while LDL traffic through the early endosomal compartment and the Golgi apparatus were unaffected. Furthermore, treatment with haloperidol notably increased the lysosomal pH and decreased the activity of lysosomal protease and β-d-galactosidase in a dose-dependent manner. We conclude that alkalinization of the lysosomes’ internal milieu induced by haloperidol affects lysosomal functionality.

## 4. Experimental Section

All chemicals were purchased from Sigma (Sigma-Aldrich Química, S.A., Tres Cantos, Madrid, Spain) unless otherwise stated.

### 4.1. Immunofluorescence Microscopy

HepG2 cells were incubated in medium with 10% LPDS supplemented with DiI-LDL (30 µg cholesterol/mL), and then incubated with no drug (control) or with 10 µM haloperidol for 16 h on glass coverslips, then fixed with 4% paraformaldehyde/PBS for 5 min. Fixed cells were permeabilized in 0.1% Triton X-100/PBS for 5 min and blocked with 2% bovine serum albumin (BSA) in PBS for 45 min. For free cholesterol staining, cells were exposed to filipin (50 mg/L in PBS) for 45 min. Cells were incubated overnight with antibodies specific for proteins of interest (LAMP-2 (Abcam, Cambridge, UK), EEA1 (BD Biosciences, San Jose, CA, USA), βCOP (ABR Affinity BioReagents, Golden, CO, USA)) or with a specific antibody against LBPA (Echelon Biosciences Inc., Salt Lake City, UT, USA), followed by incubation with Alexa fluor-conjugated IgG (Molecular Probes, Life Technologies Corporation, Carlsbad, CA, USA) in PBS at a 1:500 dilution for 45 min. Cells were mounted for microscopy and examined on a Nikon D Eclipse C1 confocal microscope.

### 4.2. Cell Culture

HepG2 hepatocarcinoma cells (ATCC HB-8065, ACTT, Rockville, MD, USA) were grown in high-glucose DMEM (Gibco, Life Technologies Corporation, Carlsbad, CA, USA) containing minimum essential medium non-essential amino acids, 10% fetal bovine serum (FBS), and antibiotics (Gibco) at 37 °C in a 5% CO_2_ atmosphere. THP-1 monocytes (ATCC TIB-202) were cultured in RPMI-1640 (Gibco) medium with l-glutamine supplemented with 0.05 mM 2-mercaptoethanol, 10% FBS and antibiotics at 37 °C in a 5% CO_2_ atmosphere. For THP-1 monocytes at 5 × 10^5^ cells/mL were treated with 1.6 × 10^−7^ M phorbol myristate acetate in RPMI-1640 with 10% FBS for 72 h to bring about differentiation; then the medium was replaced with fresh medium for a further 48 h, after which the cells were differentiated into macrophages [48]. LPDS was prepared from FBS by ultracentrifugation at 105,000× *g* for 48 h, at a density of 1.21 kg/L. For the experiments, cells were incubated in medium with 10% LPDS supplemented with LDL (30 µg/mL of cholesterol), and exposed to haloperidol dissolved in dimethyl sulfoxide (DMSO; final concentration in the medium, 0.044%) or the solvent (control). Human LDL, isolated as described previously [49], was labeled with the fluorescent probe 1,1′-dioctadecyl-3,3,3,3′-tetramethylindocarbocyanineperchlorate (DiI, Life Technologies Corporation) as described previously [50].

### 4.3. pH Measurement

Lysosomal pH on living cells was measured by a radiometric fluorescence method using the pH sensitive pHrodo-dextran and pH insensitive Alexa Fluor 488-dextran probes, as previously described [29,30]. HepG2 cells maintained in LPDS medium with LDL treated or not (control) with haloperidol (5, 10 and 25 µM) were loaded with pHrodo-dextran (0.3 µg/mL) and Alexa Fluor 488-dextran (0.3 µg/mL) (Molecular Probe) for 12 h followed by a 6 h chase to label lysosomes. Then, the cover dish was placed in a perfusion chamber (1 mL) with a continuous flux (2 mL/min) of Ringer’s solution (155 mM·NaCl, 5 mM·KCl, 2 mM·CaCl_2_, 1 mM·MgCl_2_, 2 mM·NaH_2_PO_4_, 10 mM·Hepes, 10 mM·glucose pH 7.2) and two confocal images of hundred lysosomes belonging to 6–8 selected cells were collected at 585 and 540 nm emission wavelengths by exciting first pHrodo and then Alexa Fluor probes with 568 and 488 nm lines, respectively (×20 objective; MRC-1024 and Lasersharp 5.1 software, Bio-Rad Laboratories, Barcelona, Spain). Images of blank regions of culture dish were also acquired for background subtraction. Next, the standard curve was generated for each experiment were collected from the same regions of interest after treating cells with valinomycin 20 µM and nigericin 20 µM (which collapse the membrane potential to facilitate equilibrium with external buffer) and equilibrated for 5–10 min with different calibration buffer solutions (130 mM·KCl, 1 mM·MgCl_2_, 15 mM·Hepes or 15 mM·MES) adjusted to a series of defined pH values (3.5, 4, 5, 6 and 7.2). For each experiment, the 585/540 (red/green) emission ratios for the lysosomes of each selected cell were calculated and then averaged, and radiometric data converted to pH according with the calibration curve.

### 4.4. Determination of Lysosomal Protease Activity by Flow Cytometry

DQ™ green BSA (DQ-BSA; Molecular Probes), a self-quenched green BODIPY dye conjugated to BSA, was used for measuring lysosomal protease activity. DQ-BSA requires enzymatic cleavage by protease in acidic intracellular lysosomal compartments to generate a fluorescent product that can be monitored by flow cytometry. THP1 macrophages were incubated in LPDS medium with LDL and treated or not with haloperidol for 30 min, and then DQ-BSA (10 µg/mL) was added for an additional 30 min. Green-fluorescent DQ-BSA was analyzed by flow cytometry using a FACScan-Flow cytometer (BD Biosciences). Results are expressed as specific median fluorescence intensity (MFI).

### 4.5. Determination of Lysosomal β-Galactosidase Activity by Flow Cytometry

To measure β-galactosidase activity by flow cytometry, we used the fluorogenic substrate C_12_FDG (5-dodecanoylaminofluorescein di-β-d-galactopyranoside, Life Technologies Corporation). This compound is a membrane-permeating, non-fluorescent substrate of β-galactosidase, which, after hydrolysis of the galactosyl residues, emits green fluorescence and remains confined within the cell. For the assay, cells maintained in LPDS medium with LDL were treated or not with haloperidol for 2 h before C_12_FDG (33 µM) was added, and then they were incubated for an additional 4 h. At the end of the incubation, cells were analyzed by a FACScan-Flow cytometry system (BD Biosciences). Light scatter parameters were used to exclude dead cells and subcellular debris from the analysis. The C_12_-fluorescein signal was measured on the FL1 detector and β-galactosidase activity was estimated using the MFI of the cell population.

### 4.6. Statistical Analysis

Data are shown as mean ± SEM. Means of treatment groups were compared by one-way repeated measures ANOVA followed by *post hoc* Bonferroni tests for multiple comparisons using GraphPad Prism 6 software (GraphPad Software Inc., La Jolla, CA, USA). In all cases, a confidence level of 5% was considered statistically significant (*p* < 0.05).
